# Virus-like Particles Produced in the Baculovirus System Protect Hares from European Brown Hare Syndrome Virus (EBHSV) Infection

**DOI:** 10.3390/vaccines13070731

**Published:** 2025-07-05

**Authors:** Giulio Severi, Lucia Anzalone, Laura Madeo, Anna Serroni, Claudia Colabella, Antonella Di Paolo, Pier Mario Mangili, Elisabetta Manuali, Andrea Felici, Monica Cagiola, Antonio Lavazza, Lorenzo Capucci, Giovanni Pezzotti, Antonio De Giuseppe

**Affiliations:** 1 Istituto Zooprofilattico Sperimentale dell’Umbria e delle Marche “Togo Rosati”, 06126 Perugia, Italy; l.anzalone@izsum.it (L.A.); l.madeo@izsum.it (L.M.); claudia.colabella@gmail.com (C.C.); a.dipaolo@izsum.it (A.D.P.); pm.mangili@izsum.it (P.M.M.); e.manuali@izsum.it (E.M.); a.felici@izsum.it (A.F.); m.cagiola@izsum.it (M.C.); g.pezzotti@izsum.it (G.P.); 2 Istituto Zooprofilattico Sperimentale dell’Abruzzo e del Molise “G. Caporale”, 64100 Teramo, Italy; a.serroni@izs.it; 3 Istituto Zooprofilattico Sperimentale della Lombardia e dell’Emilia Romagna “Bruno Ubertini”, 25124 Brescia, Italy; alavazza0@gmail.com (A.L.); lorenzo.capucci@alice.it (L.C.)

**Keywords:** European brown hare syndrome, EBHSV, VP60, virus-like particles, vaccine

## Abstract

**Background/Objectives:** European Brown Hare Syndrome (EBHS) is an acute and highly contagious viral disease of hares that causes considerable economic losses on wild and captive-reared hares. No preventive treatments are currently available to defeat the disease. Immunoprophylactic and biosafety measures could be applied to prevent EBHS only in captive-reared hares, where vaccination is proposed as an effective strategy. Due to the lack of a cellular substrate for virus growth, commercially available vaccines are autovaccines produced from inactivated liver suspensions of hares dead for EBHS. Therefore, using a recombinant vaccine based on VP60 major capsid protein seems a viable alternative to overcome such a problem. **Methods:** the 6xHis C-terminal tagged VP60 protein of EBHSV was expressed and produced in baculovirus, purified by affinity chromatography and the self-assembled recombinant (rEVP60-His_6_) protein. To establish the protective properties of rEVP60-His_6_-based VLPs, hares were immunised with 50 and 100 µg of VLPs and parenterally challenged with EBHSV. **Results:** all hares vaccinated with 100 µg of VLPs survived after the experimental infection, demonstrating the excellent protective ability of this prototype VLPs-based vaccine. **Conclusions:** self-assembled EBHSV rEVP60-His_6_ protein was successfully produced following a rapid, simple, low-cost protocol. Although the protective efficacy of such VLPs were experimentally demonstrated, some key aspects remain to be clarified, including the duration of protection, the entity of the antibody response, and the ability to stimulate cell-mediated response. Last, an additional aspect to be evaluated is whether the use of an adjuvant can determine whether its presence improves the performance of the recombinant VLPs vaccine.

## 1. Introduction

EBHS belongs to the complex of viral haemorrhagic diseases of lagomorphs. It is a highly contagious disease mainly affecting European brown hares (*Lepus europaeus*) and characterised by an acute course and high mortality. The disease was first described in Sweden in 1980 but recognised as a viral disease only in 1988 [1,2,3,4], and subsequently, it has spread throughout Europe [5,6,7,8,9,10]. EBHS generally occurs during autumn and winter, probably due to changing weather conditions, loss of maternal immunity, and increased densities of susceptible animals at the end of the breeding season (born-of-the-year hares). The course of the disease and the mortality depend on the hares’ density, health status, and susceptibility. During an EBHS outbreak, morbidity and mortality can reach 70–100% and 30–50%, respectively [3,11,12,13]. EBHS mainly affects adult animals, especially breeding ones; hares younger than three months become infected subclinically and acquire immunity [14,15]. The hares that survive clinical disease develop a permanent antibody response that confers protection against reinfection. In endemic areas, especially in high-density populations, the prevalence of anti-EBHSV antibodies can reach 90% [16].

EBHSV could be transmitted directly by the oro-nasal route through contact between infected and healthy animals. However, due to its resistance to environmental conditions, the transmission often occurs indirectly through food, water, and fomites contamination, including equipment, vehicles, cages, and clothing. One source of viral spread could be the carnivores (wolf, fox) and scavengers (including birds) that eat the carcasses of infected hares and then release virions with the faeces, thus contributing to virus’ environmental persistence and diffusion, even at great distances [17,18].

To date, vaccination remains the most effective method of preventing viral haemorrhagic diseases. Commercially available vaccines for RHDV are based on inactivated antigens (ERAVAC^®^ (Hipra Laboratorios S.A., Girona, Spain) and FILAVAC^® ^(Filavie S.A.S, Sevremoine, France)), myxoma viral vector (NOBIVAC^®^ Myxo-RHD, Intervet International B.V., Boxmeer, Holand), and more recently recombinant VP60 particles (YURVAC^®^ RHD (Hipra Laboratorios S.A., Girona, Spain) and FATROVAX RHD^®^ (Ozzano dell’Emilia, Bologna, Italy)), however no vaccine for EBHS has yet been marketed. In many European countries, domestic hare breeding is an important economic resource, as well as a constant source of young animals to be released into the territories for wildlife and hunting purposes. The release of healthy hares and, above all, vaccinated for EBHSV would represent an added value in terms of health management of the environment and natural territory. Moreover, this aspect prevents the spreading of disease in the wild hare population bordering the hare farms. In Italy, the available vaccine for EBHS is still an inactivated antigen-based autovaccine, which is obtained from liver suspensions of infected hares. Its efficacy is well-proven and leads to a drastic reduction in mortality rates and viral spread. In agreement with National Law D.M 17/03/1994 n. 287, its use is authorised only for established pathological and epidemiological situations (e.g., after an outbreak). Therefore, producing a recombinant vaccine based on the self-assembly of VP60 capsid protein to form VLPs is a valid alternative to the autogenous vaccine for a proper prophylactic control plan.

EBHSV is a non-enveloped, single-stranded, positive-sense RNA virus belonging to the genus *Lagovirus* of the *Caliciviridae* family, together with Rabbit Hemorrhagic Disease virus (RHDV) [19]. The genome (~7.5 kb) contains two open reading frames (ORF1 and ORF2) [20]. ORF1 encodes a ~2334 amino acids polyprotein that produces several non-structural proteins and the major capsid protein VP60 after protease cleavage; ORF2 encodes a minor structural protein (VP10). The RNA isolated from the liver of EBHSV-died hare contains two nucleic acid species: a ~7.5 kb genomic RNA (gRNA) and a 2.2 kb sub-genomic RNA (sgRNA) both, as other caliciviruses, VPg-linked at their 5′ termini [13,21]. The sgRNA contributes to producing a high level of structural proteins, which are required during the intermediate and late stages of the viral cell cycle [22]. VP60 protein, which has a molecular weight of approximately 60 kDa, self-assembles to form viral capsid and represents the main immunogenic protein. The VP60 protein of EBHSV and RHDV, like other caliciviruses, has been extensively studied for its ability to self-assemble to form Virus-Like Particles (VLPs) that are structurally and antigenically identical to wild-type infectious particles [23,24,25,26,27,28]. This protein has been successfully expressed in different eukaryotic expression systems [29,30,31,32,33,]. Moreover, VLPs of RHDV have been used to confer protection from experimental infection in immunised rabbits [34,35,36,37], and at least two vaccines based on this technology are commercially available in Europe.

In the past 20 years, VLPs have been recognised as safe and effective vaccine candidates [38,39,40,41]. The essential advantages of VLPs-based vaccines are the absence of infectivity and reversion to the virulent phenotype because they do not contain genetic material; the robust immunogenicity due to the high density of epitopes structurally exposed on the surface; and the ability to induce both humoral and cell-mediated immune response [42]. Since the early 1980s, the Baculovirus Expression System (BES) has become a versatile and robust eukaryotic expression system for producing VLPs. The ability to harvest a high amount of recombinant proteins and to perform some post-translational modifications while maintaining the biological activity of native proteins make this system suitable for the production of vaccines against viral diseases, such as EBHS.

In the present study, a prototype VLPs-based recombinant vaccine was produced in BES and easily purified using affinity chromatography. Its protective efficacy was preliminarily evaluated on hares experimentally infected with EBHSV.

## 2. Materials and Methods

### 2.1. Molecular Cloning and Generation of Recombinant Baculovirus

The VP60 encoding gene was amplified with AccuPrime Pfx DNA Polymerase (Life Technologies, Carlsbad, CA, USA) from liver samples collected during an EBHS outbreak in the Italian regions of Umbria and Marche. The VP60 gene was split into two overlapping amplicons (Table 1), cloned into the pCR^®^-Blunt Vector (ThermoFisher Scientific, Waltham, MA, USA) and sequenced (BigDye Terminator v3.1 Cycle Sequencing kit, Applied Biosystems, Foster City, CA, USA).

The two fragments obtained by enzymatic digestion were used to generate the full-length VP60 encoding gene and cloned in frame with the 6xHis tag into the baculovirus transfer vector pOET2C-6xHis (Oxford Technologies Expression, Oxford, UK).

Recombinant baculovirus was obtained by co-transfection of *Sf*21 insect cells (ThermoFisher Scientific, Waltham, MA, USA) with the plasmid containing the VP60 gene and linear bacmid DNA according to flashBAC™ system manufacturer’s instructions (Oxford Technologies Expression, Oxford, UK).

### 2.2. Production and Purification of rEVP60-His_6_ Protein

*Sf*21 insect cell line (1.3 × 10^6^ cells/mL at 27 °C) was infected with recombinant baculovirus at one MOI per cells in a protein-free medium (Insect-XPRESS™, Lonza Walkersville, Inc., Walkersville, MD, USA). Seventy-two hours post-infection, the cells were harvested, centrifuged at 400× *g* for 10 min and treated with lysis buffer (50 mM Na_2_HPO_4_/NaH_2_PO_4_ at pH 8, 0.3 M NaCl, 1% Triton X-100 and protease inhibitors). The lysate was clarified at 16,000× *g* for 30 min, and the supernatant was harvested.

Recombinant protein production was verified by an EBHS sandwich ELISA set up by the WOAH Reference Laboratory for RHD at IZSLER [43]. Briefly, the ELISA virological test is based on IgG purified from a hyperimmune serum for EBHS and from a negative serum adsorbed onto separate wells of ELISA plates. Incubation with the samples is followed by a final incubation with an anti-EBHSV MAb conjugated with peroxidase as a detection system. A sample is considered positive when the difference in OD_492_ between the IgG EBHSV positive and negative wells is >0.15. The test was performed using Sf21 cell extract infected with recombinant baculovirus expressing the rEVP60-His_6_ antigen instead of field samples and tested at different dilutions.

Sf21 cellular lysate containing the recombinant protein was purified under native conditions by affinity chromatography (His-Select^®^ Nickel Affinity Gel, Merck, Kenilworth, NJ, USA). The protein elution fractions were dialysed overnight in phosphate-buffered saline (PBS, pH 5.5) at 4 °C using Snake Skin™ Dialysis Tubing with a size of 10K MWCO (ThermoFisher Scientific, Waltham, MA, USA). The purified and dialysed VP60 recombinant protein was mixed with 4xNuPAGE sample buffer (ThermoFisher Scientific, Waltham, MA, USA) containing 10 mM DTT (Merck, Kenilworth, NJ, USA) and denatured at 99 °C for 5 min. The protein was resolved by SDS-PAGE in 10% acrylamide pre-cast NuPAGE gels and stained with SimplyBlue™ Safe Stain (ThermoFisher Scientific, Waltham, MA, USA). The EVP60-His_6_ recombinant protein was quantified by the Bradford method (Bio-Rad Laboratories, Hercules, CA, USA) and filtered with a 0.22 µm filter (Minisart^®^, Sartorius, Göttingen, Germany).

### 2.3. Transmission Electron Microscopy

To confirm the presence of VLPs, purified EVP60-His_6_ was adsorbed onto 400 MESH copper grids (QUANTIFOIL^®^ Micro Tools GmbH, Jena, Germany) for 4 min. The sample was negatively stained with a 2% NaPT solution (Merck, Kenilworth, NJ, USA) at pH 6.9 for 1 min. Excess fluid was removed with blotted paper, and the sample was left to air dry. The grids were observed with a CM12 STEM electron microscope (Philips, Eindhoven, The Netherlands) operating at 80 kW. VLPs were identified based on their morphological characteristics.

### 2.4. Experimental Procedure

#### 2.4.1. Virus Preparation

The viral suspension used for the challenge was obtained from a pool of livers of EBHS-died hares. Briefly, livers (50 g) were homogenised in sterile saline solution (200 mL) for three cycles at 8000 rpm for 60 s using Ultra Turrax (Merck, Kenilworth, NJ, USA). The homogenate was centrifuged at 12,000× *g* for 15 min, and the supernatant was filtered with a 0.22 µm filter (Minisart^®^, Sartorius, Göttingen, Germany). All steps were performed at +4 °C.

#### 2.4.2. Immunisation Protocol and Challenge

Fifteen eight-week-old hares were housed in a Biosafety Level 3 area, tested for the absence of specific anti-EBHSV antibodies and randomly divided into three groups. Seven, three and five hares were immunised, respectively, via subcutaneous injection with 100 μg of VLPs, 50 μg of VLPs and PBS without adjuvant (control group). All the animals were inoculated twice in three-week intervals and challenged intramuscularly with 1 mL of EBHSV suspension three weeks after the second immunisation. The animals were observed daily for clinical signs and changes in feeding rates and behaviour were recorded. Finally, surviving animals were suppressed, according to D. Lgs n. 26/2014 authorisation.

### 2.5. Laboratory Analysis

#### 2.5.1. Blood Sampling, Serological and Virological Assay

Blood samples were collected at 0, 3, 6, 8 and 10 weeks after the first immunisation and stored at −20 °C. All the collected sera were diluted 1:10, and the detection of specific anti-EBHSV antibodies was performed using a competitive ELISA set up at the WOAH Reference Laboratory for RHD at IZSLER [14,43,44].

Liver tissue samples were collected and analysed for the presence of EBHSV antigen using an EBHS sandwich ELISA (IZSLER, Brescia, Italy), using again the reference method as reported above [43].

#### 2.5.2. Histological Examination

All the tested hares were subjected to necropsy immediately after their death or at the end of the experiment when the survivors were humanely sacrificed (four weeks after challenge). Their organs (liver, trachea, lung, kidney and spleen) were collected to evaluate EBHS characteristic pathological lesions. Briefly, the organs were fixed in 10% buffered formalin and embedded in paraffin wax. Four µm thick sections were stained with Hematoxylin and Eosin (HE) (Histo Line Laboratories, Milan, Italy). Images were digitised using the microscope Eclipse Ci-L (Nikon Corporation, Tokyo, Japan) using NIS-Elements Br-2 as software (v 5.10; Nikon).

#### 2.5.3. Molecular Assay

Total RNA was extracted from 5 mg of the collected organs (liver, trachea, lung, kidney and spleen) using RNAeasy^®^ Micro kit (Qiagen, Hilden, Germany) following the manufacturer’s instructions. Five µL of extracted RNA was reverse transcribed using SuperScript™ II Reverse Transcriptase (ThermoFisher Scientific, Waltham, MA, USA) following the manufacturer’s instructions. VP10 region was subject to PCR and Real-Time PCR and amplified with the following primers: EBH-RTF 5′-TTATGGCGGTTGCGTCGCGC-3′/EBH-RTR5′-TTCCCACGAGGTCCGTAAAGAAGC-3′ using the FAM/TAMRA 5′-CGACTTCAGTCTTACC-3′ probe and TaqMan™ Fast Advanced Master Mix for qPCR (ThermoFisher Scientific, Waltham, MA, USA).

### 2.6. Statistical Analysis

Hares immunised with 100 μg of VLPs and hares inoculated with PBS (control group) were compared through Fisher’s exact test, where 95% confidence intervals were also calculated. A *p*-value < 0.05 was considered significant.

## 3. Results

### 3.1. Production of VLPs

Expression and yield of rEVP60-His_6_ protein production in Sf21 insect cells infected with the corresponding recombinant baculovirus was verified by virological ELISA and SDS-PAGE. The ELISA titration of the recombinant protein sample was still positive at a dilution of 1/5000, demonstrating its high production and significant similarity to the antigenic profile of EBHSV (Table 2).

**Figure 1:**
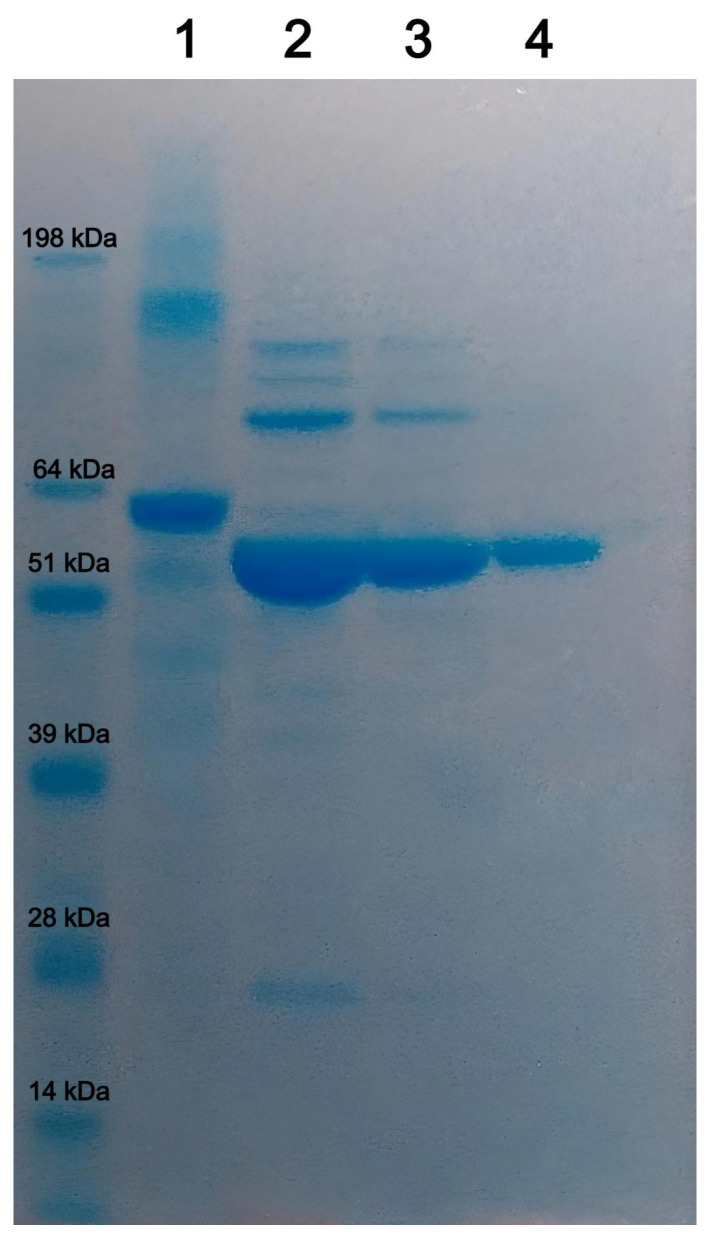
SDS-PAGE and Coomassie gel staining of purified rEVP60-His_6_ protein; recombinant protein elution fractions (lanes 2–4); bovine serum albumin—2 μg (lane 1); protein standard molecular weight is reported.

Coomassie gel staining of purified rEVP60-His_6_ protein showed the presence of a colourimetric band with the expected size of ~60 kDa (Figure 1).

As shown in Figure 1, a high recovery and degree of purity of rEVP60-His_6_ protein was obtained when the nickel affinity chromatography was carried out under native conditions.

**Figure 2:**
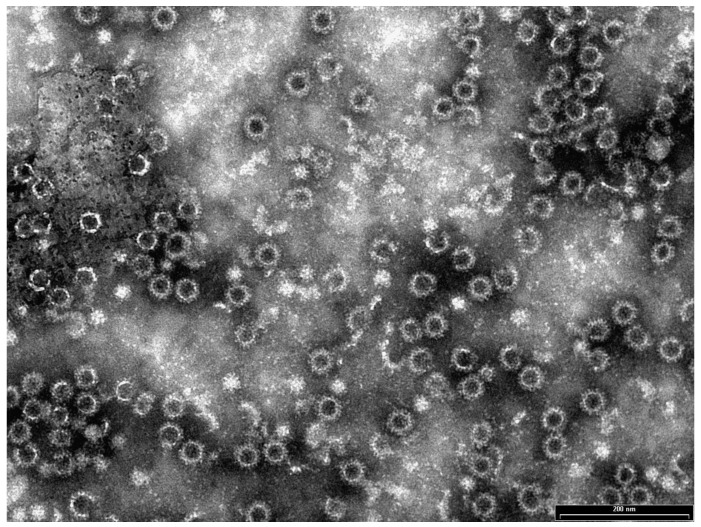
TEM images of rEVP60-His_6_ protein self-assembly to form VLPs (bars = 200 nm).

In order to verify the ability of the VP60 recombinant protein to self-assemble to form VLPs, the elution fractions were subjected to dialysis. Preliminary investigation by electron microscopy of rEVP60-His_6_ protein dialysed at different pHs (7, 8 and 9.5) did not reveal the presence of VLPs. The assembly conditions were improved when elution fractions containing the highest amounts of rEVP60-His_6_ were subjected to dialysis at a slightly acidic pH (Figure 2). In this condition, many VLPs were observed in terms of size, quantity and morphology. The quantification of purified VLPs by the Bradford method showed a final yield of approximately 2.4 mg for 10^9^ insect cells, and the immunising doses were finally formulated without adjuvant.

### 3.2. Experimental Infection of Hares

**Figure 3:**
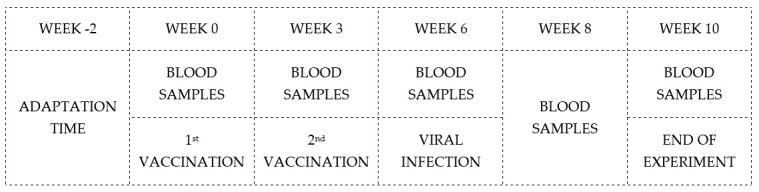
Schematic representation of phases and timing of experimental protocol.

Only one of the hares selected for the experiment was found slightly positive for the competitive ELISA, which could result either from regressive contact with the virus or, more likely, as a residue of maternal antibodies. However, this hare was serologically negative before the first immunisation. The schematic timeline of the main phases is illustrated in Figure 3.

#### 3.2.1. Control Group

Three out of five hares (93M, 95F and C7) died about 96 h after the experimental EBHSV infection. A consistent and drastic reduction in food consumption and a decrease in stimuli response were simultaneously observed. The main necropsy findings related to EBHSV infection in these animals were diffuse haemorrhages in the trachea, lungs, and liver. The latter appeared congested and enlarged, with several diffuse haemorrhagic foci in the visible lobules. Finally, the spleen appeared enlarged (Figure S1).

As shown in Table 3, laboratory analysis confirmed the presence of EBHSV both in the target organ (liver) by ELISA and in other organs by direct detection of the viral genome by molecular methods.

**Figure 4:**
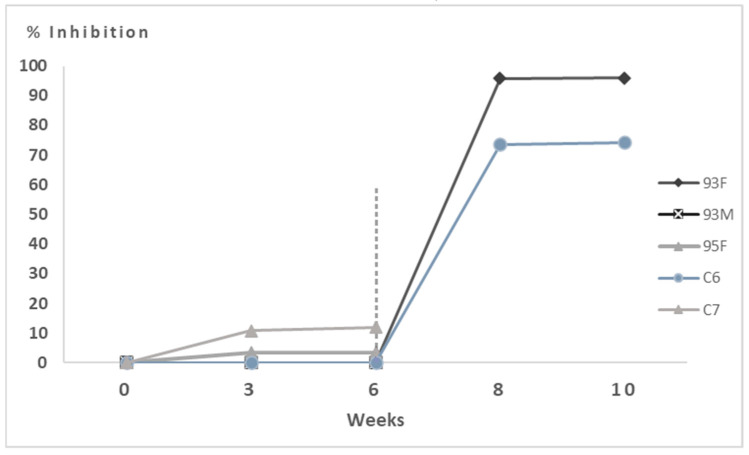
Competitive ELISA assay performed on unvaccinated hares (control group). OD values were measured in duplicate and recorded at OD_492_ nm. Sixth week challenge time point is visualised by the vertical dashed line.

Conversely, direct detection assays performed on the two hares (C6 and 93F) that survived experimental EBHSV infection and sacrificed at the end of the experiment resulted negative, except of a slight positivity detected by Real-Time PCR in the liver of the 93F hare. Moreover, indirect serological tests by competitive ELISA showed a good level of seroconversion at 14 days post-infection (dpi) (Figure 4).

#### 3.2.2. Hares Immunised with 50 μg of VLPs

Two out of three hares (91MB and 95M) died, respectively, at 4 and 9 dpi, and a gradual decline, in general, clinical condition was observed. At the necroscopy examination, hare 95M showed tracheal, pulmonary and hepatic haemorrhages together with severe diffuse haemorrhages in both gastric and enteric areas with severe abdominal viscera congestion (Figure S2). Hare 91MB autopsy displayed tracheal and hepatic haemorrhages and a reduced consistency of the kidneys. The finding of this dead animal in the cage, near the corner of the den, might indicate the possibility of a traumatic death origin not related to experimental EBHSV infection. This could explain the virological negativity by suggesting the animal could survive the experimental infection if the same animal had remained alive.

**Figure 5:**
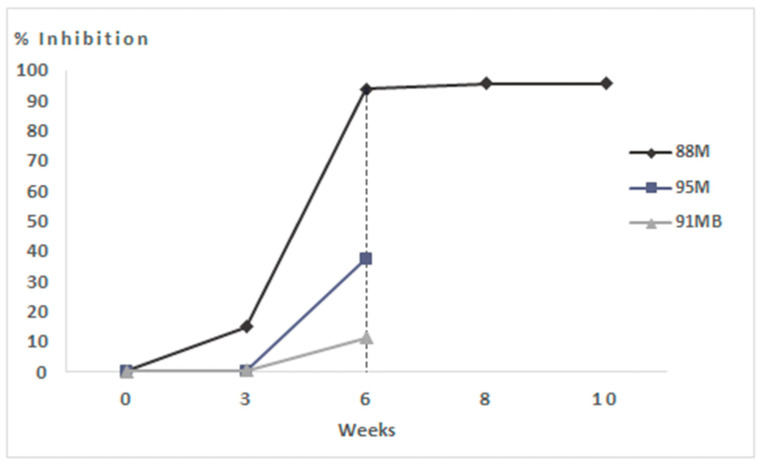
Competitive ELISA assay performed on hares immunised with 50 μg of VLPs. OD values were measured in duplicate and recorded at OD_492_ nm. Sixth week challenge time point is visualised by the vertical dashed line.

Indeed, both hares tested negative on the virological ELISA assay, while the molecular investigation was positive only on the spleen and slightly positive on the kidney for the 95M hare (Table 3), and both showed weak vaccine-induced seroconversion (Figure 5).

Conversely, the hare that survived the challenge (88M) was the only one to respond to the vaccine with marked seroconversion after booster (Figure 5). Indeed, when this animal was humanly sacrificed at the end of the experiment (four weeks post-infection), a very low Real-Time PCR positivity (Ct 39 and Ct 38 for the liver and trachea, respectively) was detected (Table 3), testifying to a likely abortive viral replication.

#### 3.2.3. Hares Immunised with 100 μg of VLPs

All seven hares immunised with 100 µg of VLPs survived the challenge without showing any clinical signs and anatomopathological lesions related to EBHSV infection (Figure S3). Moreover, the excellent clinical response shown in the hours, days and weeks following experimental infection up to week 10 (28 dpi) was propaedeutic to confirm the efficacy of the prototype VLP-based vaccine.

**Figure 6:**
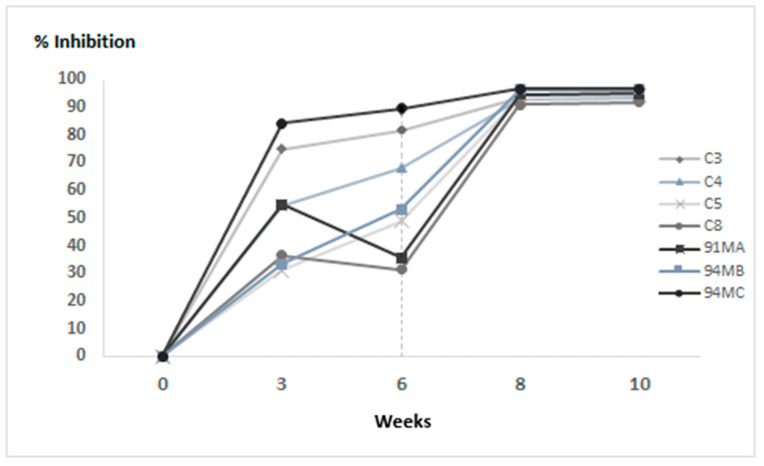
Competitive ELISA assay performed on hares immunised with 100 μg of VLPs. OD values were measured in duplicate and recorded at OD_492_ nm. Sixth week challenge time point is visualised by the vertical dashed line.

Post-mortem laboratory analysis showed that it was not possible to detect EBHSV by ELISA in the target organ except for one animal (C3) and, at the same time, in no organ by direct detection of the viral genome. In contrast, Real-Time PCR showed the weak presence of nucleic acid in the spleen of the C4 hare in the absence of viral antigen (Table 3). The indirect serological assay showed a good antibody response already after the first administration of VLPs for most animals and a high antibody titer until the end of the trial (Figure 6). Furthermore, the excellent immunising properties of the prototype vaccine are highlighted by comparing the serological response between the different groups of animals during the immunisation protocol (Figure S4).

#### 3.2.4. Histopathology

The main histopathological findings were observed in the trachea and liver; the severity of injuries varied between experimental groups. Briefly, in the control group, necrosis of lining epithelial cells with loss of microvilli, edoema and submucosal lymphoplasmacytic tracheitis, and vessel congestion were found. The liver parenchyma appeared degenerated, with multifocal foci of hepatocyte necrosis, hemorrhagic and severely congested. In addition, histological examination showed diffuse acute necrosis of the periportal spaces and the intermediate area, diffuse splenic necrosis, vascular congestion and haemorrhages (Figure S5).

In the vaccinated group (50 µg), tracheal lesions were less severe, with focal necrosis of the lining epithelial cells, mild inflammatory reaction and moderate vessel congestion. Occasionally, neutrophilic exudation was detected in the lumen (Figure S6). However, the histological examination findings reported above do not completely agree with the laboratory results, as can be highlighted in Table 3.

In the vaccinated group (100 µg), tracheal mucosa appeared well preserved, with the presence of numerous microvilli, goblet cells and Mott cells, indicating immunoglobulins production (Figure S7). The liver parenchyma was moderately congested.

### 3.3. Statistical Analysis

Evaluation of the efficacy of the prototype vaccine was calculated only for animals immunised with 100 µg of VLPs compared with the control group by Fisher’s exact test.

In the treated group (*n* = 7) survival rate was 100% (IC95%: 65–100), while in the untreated group (*n* = 5), it was 40% (IC95%: 12–77); the difference was statistically significant (*p*-value = 0.045).

## 4. Discussion

European brown hare syndrome (EBHS) is a highly contagious acute viral disease that predominantly affects the European brown hare (*Lepus europaeus*) and is widespread throughout Europe [2,5,7,14]. In terms of clinical manifestations, EBHS is almost identical to rabbit hemorrhagic viral disease (RHDV) with typical lesions, e.g., diffused haemorrhages and liver necrosis, and is characterised by high mortality [1,14]. An important contribution to disease control is vaccine prophylaxis. However, to date, no registered vaccine is commercially available in Italy, only inactivated antigen-based autogenous vaccine could currently be used in restricted situations.

VP60 protein of both EBHSV and RHDV has been successfully produced in several expression systems, and VLPs of RHDV have conferred protection against challenge with lethal strains [33,35]. Unlike conventional inactivated vaccines, VLPs display a good safety profile and highly efficient induction of humoral and cellular immune responses [31,36]. In the present study, EBHSV capsid protein VP60 was expressed and produced in BES and its self-assembly properties were also evaluated. Preliminary investigations focused on evaluating the expression level of rEVP60-His_6_ protein produced in *Sf*21 insect cells and obtaining the best conditions for its production and purification. Through the in-frame insertion of a 6xHis tag into the C-terminal portion of the VP60 encoding gene, it was possible to rapidly and easily purify the protein by affinity chromatography with good quantitative and qualitative yields. Also, the results indicated that the rEVP60-His_6_ protein showed a similar antigenicity to the wild-type VP60 protein.

To verify the ability of the purified rEVP60-His_6_ antigen to self-assemble and form VLPs, several investigations using different pH were performed. The dialysis of the recombinant protein under neutral or slightly alkaline pH conditions did not reveal the presence of VLPs. Conversely, rEVP60-His_6_-VLPs self-assembled under slightly acidic pH conditions (5.5) and showed similar morphology and size than native EBHSV. The results obtained by transmission electron microscopy confirmed the excellent self-assembly properties of rEVP60-His_6_ antigen.

Evaluation of the efficacy of rEVP60-His_6_ protein as a prototype VLPs-based vaccine was carried out through a pilot study on hares experimentally infected with EBHSV. A total of fifteen hares were involved in the study, of which five were inoculated with PBS (control group), three were immunised with 50 µg of VLPs, and seven were immunised with 100 µg of VLPs. A 60% mortality rate was observed in the control group 96 h after the challenge, confirming the pathogenicity of the viral field strain intramuscularly inoculated and in line with the epidemiological data reported [18]. All laboratory analyses were consistent with what was clinically observed. In fact, both virological and molecular tests confirmed the presence of the virus in the EBHSV-dead hares (Table 3). EBHSV-related lesions were also observed with anatomopathological and histological examination (Figures S1 and S5). Conversely, the experimental infection with EBHSV induced a marked humoral immune response in the two surviving hares.

The results obtained after the immunisation of hares with 50 µg of VLPs were difficult to interpret. Although lesions were found on necropsy and histological examination (Figures S2 and S6), virological tests on the target organ were negative. Moreover, hare 95M, which showed weak seroconversion after booster, probably died for EBHSV. In fact, the viral genome detection yielded positive results by Real-Time PCR in the spleen (Ct 32) and faintly detectable in the liver (Ct 39), in contrast to what was expected. Such a situation is typical of the chronic, prolonged course of the disease when the viral clearance occurs mainly in the splenic reticuloendothelial cells. This justifies the recommendation to always test the spleen, besides the liver, to diagnose EBHS. In fact, the frequency of chronic course of EBHS in hares is much higher than in rabbits infected with RHDV [43,44]. The 91MB and 88M hares showed negative results for EBHSV, but the latter was the only one of that group who responded to the VLPs-based vaccine with a consistent seroconversion after booster and survived the challenge. This situation could be due to the low antigen dose that caused insufficient seroconversion to prevent EBHS infection following challenge by the intramuscular route.

All hares immunised with 100 µg of VLPs showed good seroconversion already after the first administration and high antibody titer until the end of the trial. These results suggest that EBHSV VLPs can provide protection against EBHSV. Moreover, no EBHSV was detected by direct detection of the viral genome, which further confirms that VLPs can induce a strong immune response while also inhibiting virus replication and spread. The only exception was in the C3 hare that tested positive on the ELISA antigen test, which could be considered a false positive given the negativity found on all organs in the direct molecular assay and the absence of anatomopathological and histological lesions compatible with EBHSV (Figures S3 and S7). The prototype VLPs-based vaccine in the immunisation dosage of 100 µg protected hares from experimental infection with EBHSV, as all animals survived the challenge by showing excellent overall clinical scores up to 28 dpi. This result indicates that the vaccination had a statistically significant influence on the survival of hares for this treated group (*p*-value = 0.045).

Furthermore, in all hares vaccinated with 100 µg of VLPs, it was possible to prevent the onset of severe disease. These results suggest that the VLP vaccine prototype is also able to confer protection against infection. However, viral circulation cannot be excluded for this group of hares because the difficult management procedures and housing of these animals did not allow for the collection of oro-nasal swabs, i.e., a likely way of viral excretion, during weeks 6–10. Therefore, to date, it is not possible to confirm that the animals never became virologically positive, perhaps transiently and mildly. Moreover, the clinical scores observed suggest the absence of viral circulation in animals vaccinated with 100 µg of VLPs. Finally, these results clearly indicate that this prototype VLPs-based vaccine can represent a viable alternative to the use of conventional autogenous vaccines.

## 5. Conclusions

In this study, EBHSV rEVP60-His_6_ protein was successfully produced, purified, and self-assembled to form VLPs following a simple, rapid, and low-cost protocol. The main results obtained were the evaluation of the protective efficacy of such synthetic antigen assembled into VLPs. At a dose of 100 µg, this recombinant protein was able to protect hares from disease and almost certainly from infection, as experimentally demonstrated. Despite these promising results, some key aspects remain to be clarified, including the duration of protection, the entity of the antibody response, the ability to stimulate cell-mediated response, and the capacity to protect hares after challenge through natural way of infection (oro-nasal), especially compared with the autogenous vaccine.

An additional aspect to be evaluated is whether an adjuvant can be used in the vaccine formulation to determine whether its presence improves the performance of the recombinant VLPs vaccine even at doses below 100 µg. A field trial could verify all these aspects and confirm the full functionality of the prototype VLPs-based vaccine by proposing it definitively as a real and concrete alternative to the traditional autogenous vaccine.

## 6. Patents

Italian patent No. 102017000091210; European patent n° EP3665185 B1.

## Figures and Tables

**Table 1:** Oligonucleotides used in this study.

Sequence 5′-3′	Primer
GATAGTCTCGAGGCCACCATGGAGGGTAAGCCTCGGGCTG	EBHS-VP60-F
GAGCCCGACAATTGGTGCACC	EBHS-VP60/ApaLI-R
CAGACAACAGGTGGGGTGCAC	EBHS-VP60/ApaLI-F
CCTAGGCCGGCGACATAGGAATATCCAGTGGT	EBHS-VP60/NgoMIV-R

**Table 2:** rEVP60-His_6_ protein expression on the Sf21 cells infected with the recombinant baculovirus performed by the virological ELISA. The values are expressed as OD_492_ nm.

Cellular Lysate		
Dilution	Positive IgG	Negative IgG
1:5	2.9	0.25
1:50	2.57	0.16
1:500	1.48	0.157
1:5000	0.361	0.182

**Table 3:** Laboratory analysis performed on collected organs from unvaccinated (control group) and vaccinated hares. Real-Time PCR positive control: Ct 22 for liver; Ct 23 for spleen and lung; Ct 21 for kidney and trachea.

Group	Life Outcome	Animal	Virological Assay (L)	PCR	Real-Time PCR
**Control**	Dead	93M	(+)	(+) L, S, K, T	(+) L (Ct 20), S (Ct 15), K (Ct 23), T (Ct 22)
	Dead	95F	(+)	(+) L, S, Lu, K, T	(+) L (Ct 25), S (Ct 21), Lu (Ct 32), K (Ct 31), T (Ct 31)
	Dead	C7	(+)	(+) L, S, Lu, K, T	(+) L (Ct 23), S (Ct 22), Lu (Ct 29), K (Ct 26), T (Ct 33)
	Survivor	93F	(-)	(-)	(±) L (Ct 37)
	Survivor	C6	(-)	(-)	(-)
**Vaccinated** (50 µg VLPs)	Dead	95M	(-)	(+) S	(+) S (Ct 32); (±) K (Ct 38)
	Dead	91MB	(-)	(-)	(-)
	Survivor	88M	(-)	(-)	(±) L (Ct 39), T (Ct 38);
**Vaccinated** (100 µg VLPs)	Survivor	91MA	(-)	(-)	(-)
	Survivor	94MB	(-)	(-)	(-)
	Survivor	94MC	(-)	(-)	(-)
	Survivor	C3	(+)	(-)	(-)
	Survivor	C4	(-)	(-)	(±) S (Ct 38)
	Survivor	C5	(-)	(-)	(-)
	Survivor	C8	(-)	(-)	(-)

**Legend:** (+) positive; (±) slightly positive; (-) negative; (Ct) threshold cycle; L (liver); S (spleen); Lu (lung); K (kidney); T (trachea).

## Data Availability

The data are available upon request.

## Supplementary Materials

The following supporting information can be downloaded at https://www.mdpi.com/article/10.3390/vaccines13070731/s1, Figure S1: Necropsy analysis of unvaccinated hares (control group). (a) Multifocal necrosis and diffused haemorragic foci in the liver. (b) Massive spleen enlargement; Figure S2: Necropsy analysis of hares vaccinated with 50 μg of VLPs. (a) Multifocal necrosis and diffused haemorragies in the liver. (b) Spleen enlargement; Figure S3: Necropsy analysis of hares vaccinated with 100 μg of VLPs. (a) Absence of macroscopically apparent lesions. (b) Spleen normal in size and macroscopic appearance.; Figure S4: Comparison of mean values of the percentage inhibition of the indirect serological test between the vaccinated and unvaccinated hares groups. The bars indicate the Standard Deviation; Figure S5: Histological examination of the control group. (a) Multifocal necrosis of the respiratory epithelium (arrow), oedema and inflammation of the submucosa (asterisk). (b) Fatty degeneration of hepatocytes (arrowhead) and multiple foci of hepatocyte necrosis (arrow). Haematoxilin and Eosin (HE). Bars 500 μm; Figure S6: Histological examination of hares immunised with 50 μg of VLPs. (a) Focal necrosis of epithelial cells (arrow) and neutrophilic exudate in the lumen (asterisk). (b) Diffuse haemorrhages and liver vascular congestion. Haematoxilin and Eosin (HE). Bars 500 μm; Figure S7: Histological examination of hares immunised with 100 μg of VLPs. (a) The tracheal mucosa appears normal, with the presence of goblet cells (arrow) and Mott cells (arrowhead). (b) Mild vascular congestion of the liver. Haematoxilin and Eosin (HE). Bars 500 μm.
