# Supplementary material for: Virus-like Particles Produced in the Baculovirus System Protect Hares from European Brown Hare Syndrome Virus (EBHSV) Infection
Source: Vaccines (Basel). 2025 Jul 5;13(7):731. doi: 10.3390/vaccines13070731 (PMC12299225; doi:10.3390/vaccines13070731)
Supplement: Supplementary file 1 [file vaccines-13-00731-s001.zip › vaccines-3667201-supplementary.pdf]

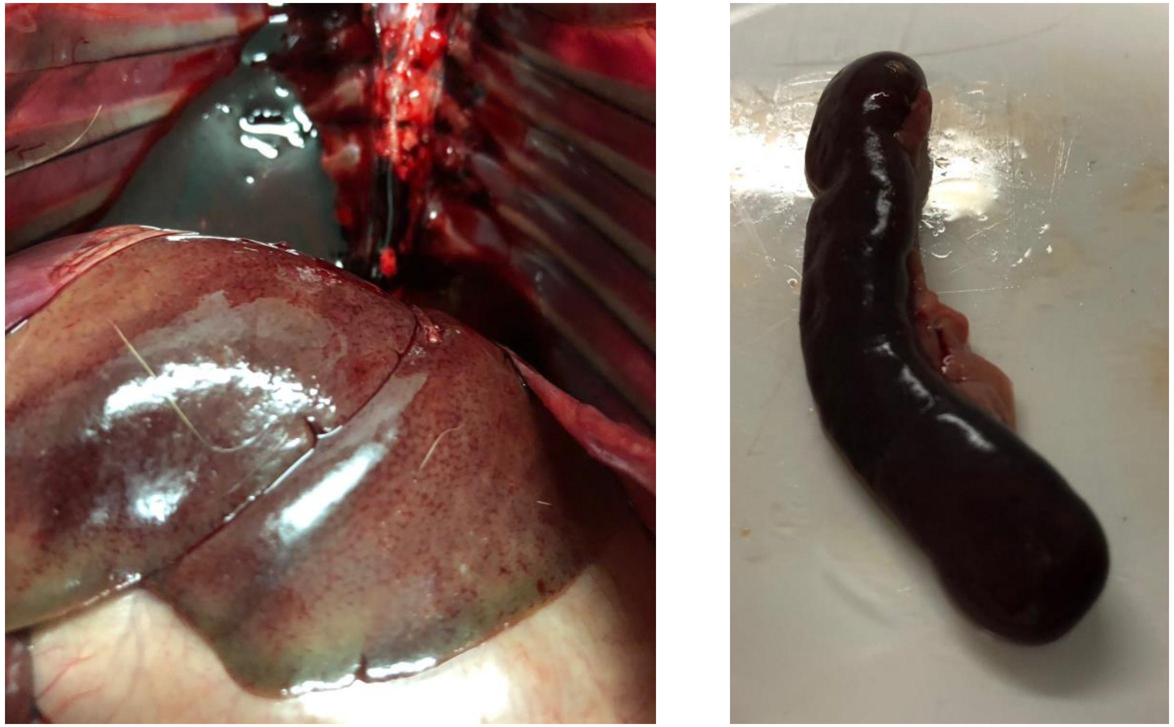

(**a**) (**b**)

Figure S1. Necropsy analysis of unvaccinated hares (control group). (a) Multifocal necrosis and diffused haemorragies in the liver. (b) Massive spleen enlargement.

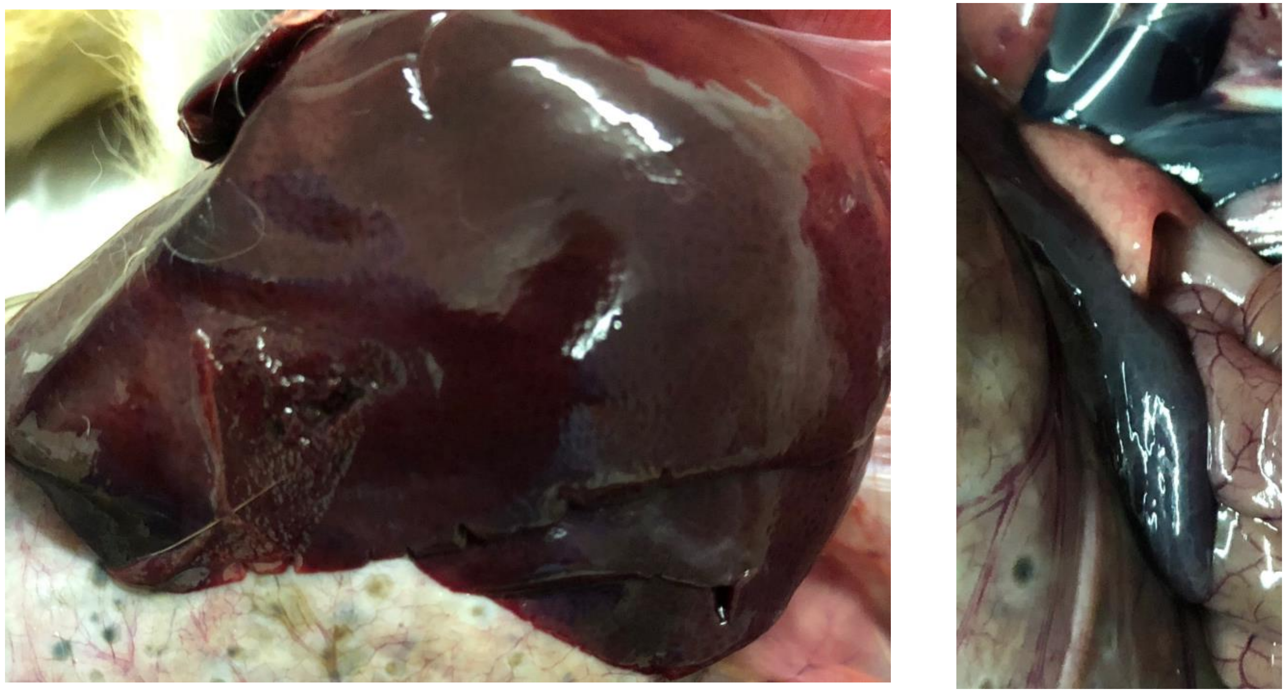

(**a**)

(**b**)

Figure S2. Necropsy analysis of hares vaccinated with 50 µg of VLPs. (**a**) Multifocal necrosis and diffused haemorragies in the liver. (b) Spleen enlargement.

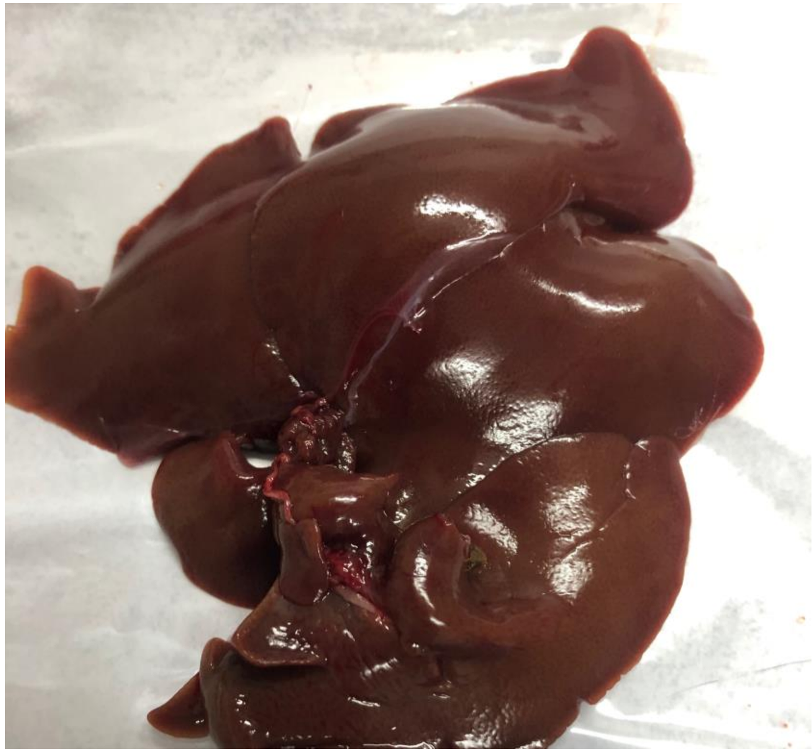

(**a**)

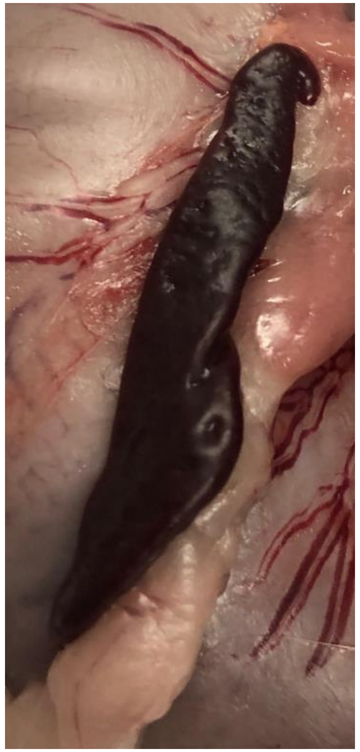

(**b**)

Figure S3. Necropsy analysis of hares vaccinated with 100 μg of VLPs. (a) Absence of macroscopically apparent lesions. (b) Spleen normal in size and macroscopic appearance.

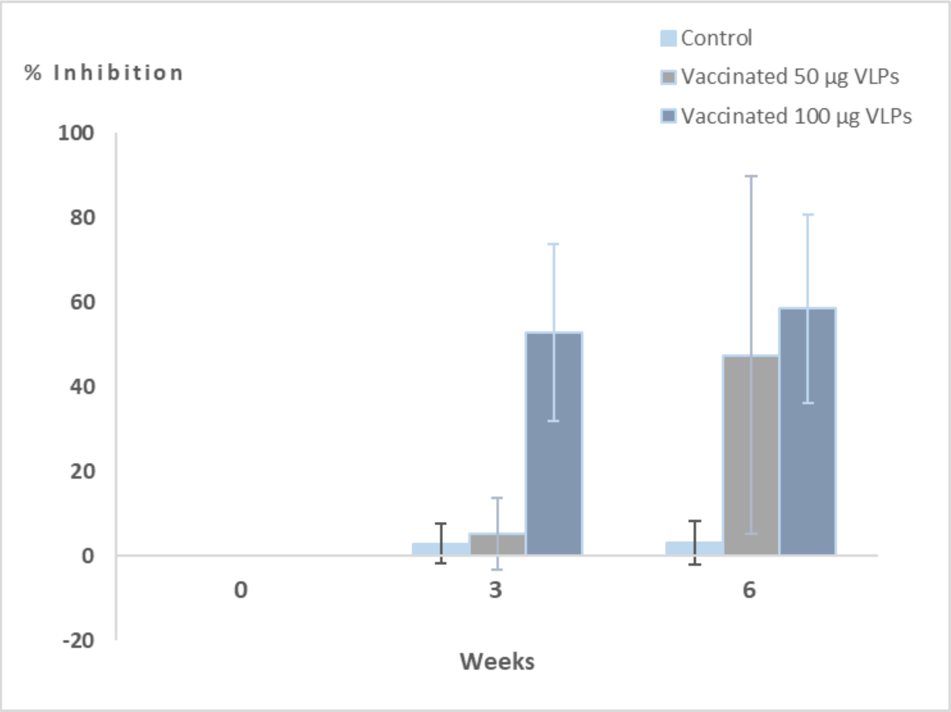

Figure S4. Comparison of mean values of the percentage inhibition of the indirect serological test between the vaccinated and unvaccinated hares groups. The bars indicate the Standard Deviation.

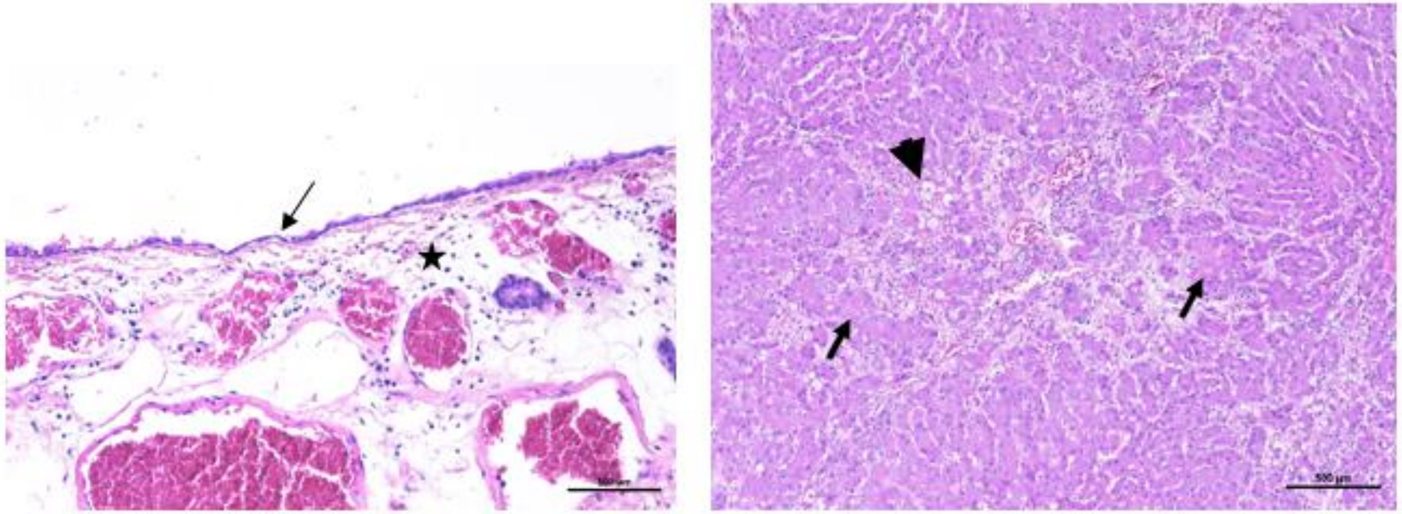

(**a**) (**b**)

Figure S5. Histological examination of the control group. (**a**) Multifocal necrosis of the respiratory epithelium (arrow), edema and inflammation of the submucosa (asterisk). (b) Fatty degeneration of hepatocytes (arrowhead) and multiple foci of hepatocyte necrosis (arrow). Haematoxilin and Eosin (HE). Bars 500 μm.

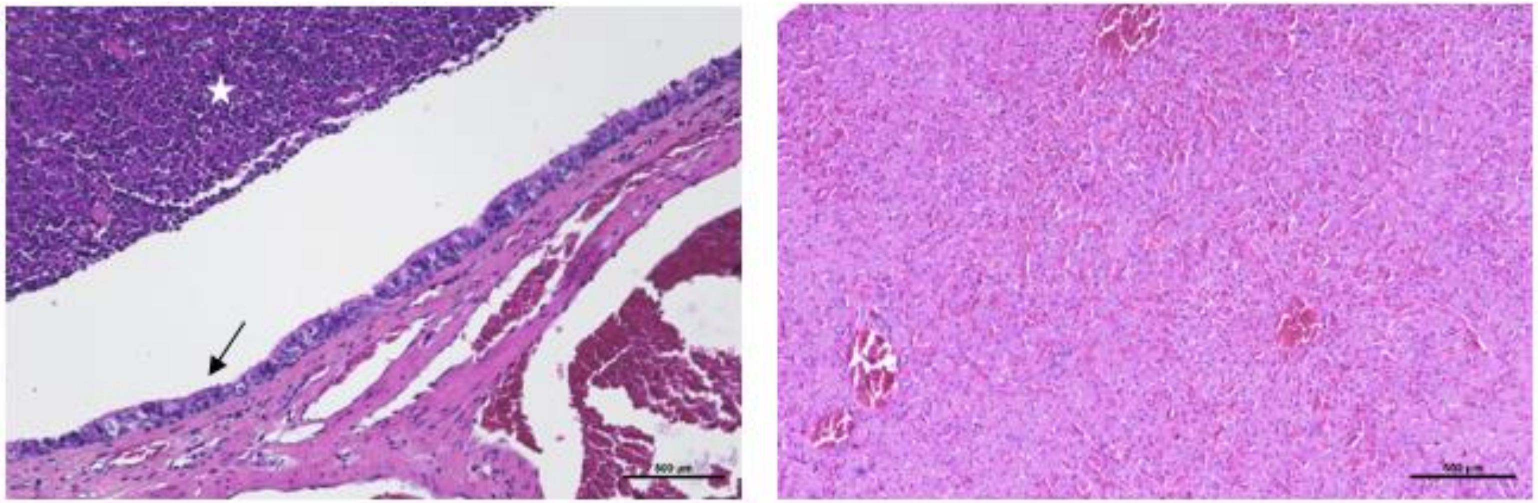

(**a**) (**b**)

Figure S6. Histological examination of hares immunised with 50 µg of VLPs. (**a**) Focal necrosis of epithelial cells (arrow) and neutrophilic exudate in the lumen (asterisk). (**b**) Diffuse haemorrhages and liver vascular congestion. Haematoxilin and Eosin (HE). Bars 500 µm.

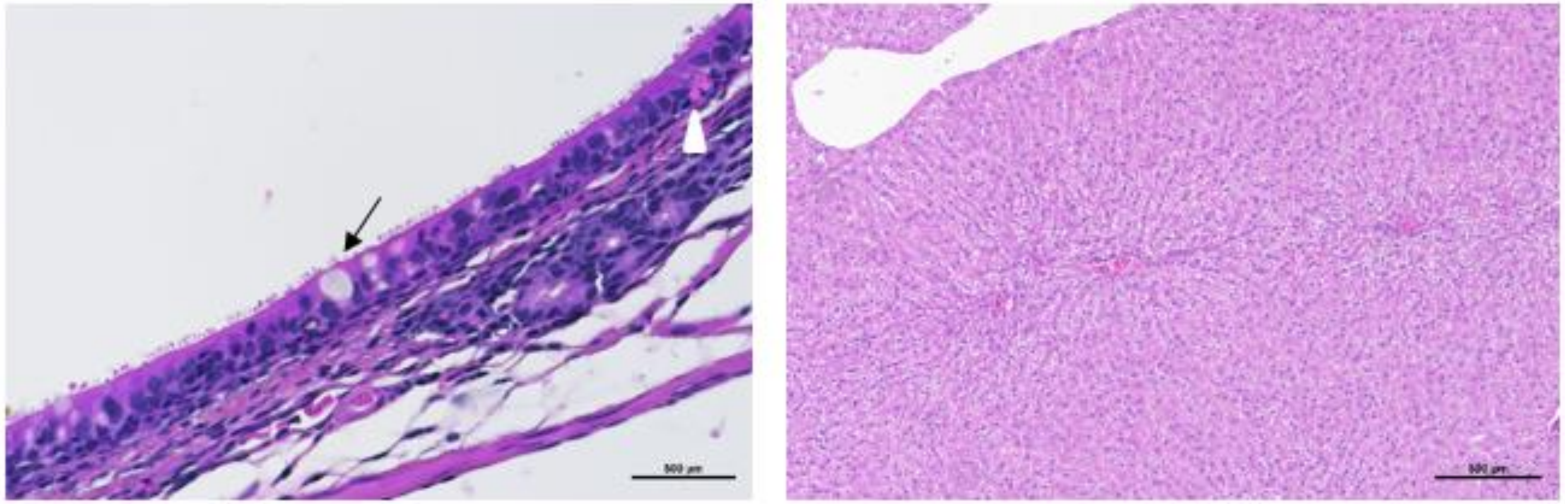

(**a**) (**b**)

Figure S7. Histological examination of hares immunised with 100 µg of VLPs. (**a**) The tracheal mucosa appears normal, with the presence of goblet cells (arrow) and Mott cells (arrowhead). (**b**) Mild vascular congestion of the liver. Haematoxilin and Eosin (HE). Bars 500 µm.
